# ON/OFF Phenomenon in 4‐Aminopyridine Therapy in Spinocerebellar Ataxia 27B: Therapeutic and Diagnostic Insights

**DOI:** 10.1002/mdc3.70692

**Published:** 2026-05-28

**Authors:** Chiara Caneda, Eleonora Bertoncello, Alessia Peghin, Sara Satolli, Filippo Maria Santorelli, Michele Tinazzi, Ilaria Antonella Di Vico

**Affiliations:** ^1^ Neurology Unit, Department of Neurosciences, Biomedicine and Movement Sciences Policlinico Borgo Roma, University of Verona Verona Italy; ^2^ Division of Neurology and Neurorehabilitation, Department of Neurosciences and Rehabilitation Santobono Pausilipon Children's Hospital (AORN Santobono Pausilipon) Naples Italy; ^3^ Molecular Medicine for Neurodegenerative and Neuromuscular Diseases Unit IRCCS Stella Maris Foundation Pisa Italy

**Keywords:** 4‐aminopyridine, SCA27B, spinocerebellar ataxia 27B

Spinocerebellar ataxia type 27B (SCA27B) is a recently characterized genetic entity caused by a heterozygous pathogenic GAA repeat expansion in the FGF14 gene. It is increasingly recognized as a frequent cause of autosomal dominant late‐onset cerebellar ataxia (ADCA), accounting for a substantial proportion of cases formerly considered genetically unexplained, with recent data from Italian cohorts reporting rates up to 38% among ADCA cases.^1^ The phenotype typically includes adult‐onset cerebellar ataxia, episodic gait instability, and downbeat nystagmus, often with a fluctuating course.^2^ Despite the limited therapeutic options available, 4‐aminopyridine (4‐AP), a selective voltage‐gated potassium channel blocker, has demonstrated symptomatic benefit in patients with SCA27B, including improvements in clinical symptoms and gait performance.^3^, ^4^

We report the case of a 77‐year‐old man with a negative family history and genetically confirmed SCA27B (*FGF14* expansion: 265 ± 2 GAA repeats) who experienced a clear and reproducible acute improvement in motor symptoms following administration of 4‐AP. An immediate‐release galenic formulation of 4‐AP was used, as extended‐release formulations were not readily available in our setting.

Over the prior 5 years, the patient had progressively developed imbalance, gait ataxia, limb incoordination, slow saccadic eye movements, downbeat nystagmus and slurred speech. The symptoms showed a typical diurnal fluctuation, with worsening in the morning and after physical exertion. No clear “symptom‐free” intervals were reported since disease onset, and no association with alcohol or caffeine intake was identified. He also reported a left frontoparietal headache upon awakening, partially relieved by rest. Before treatment, he could barely walk a few meters even with bilateral support. A brief attempt with acetazolamide was discontinued due to poor tolerability (fatigue, confusion).

A therapeutic trial was initiated with an oral galenic formulation of 4‐AP at a starting dose of 4 mg/day, which was subsequently titrated to 4 mg four times daily. From the first doses, he reported a striking improvement within 40 minutes, with better gait (walking 20 meters with rollator), reduced oscillopsia and less speech difficulty during exertion. The benefit was most pronounced on gait disturbances and speech and during the first 3–4 hours post‐dose, disappearing when the drug was omitted with recurrence of slurred speech, limb incoordination, cognitive slowing and headache (Fig. 1). The medication was gradually titrated up to four times per day, reducing fluctuations after each intake, with symptom persistence mainly upon awakening. A structured diary and video documentation confirmed the reproducibility of this on–off phenomenon, with daily fluctuations modulated by fatigue and stress (Videos 1, 2, 3, 4, 5).

**Figure 1:**
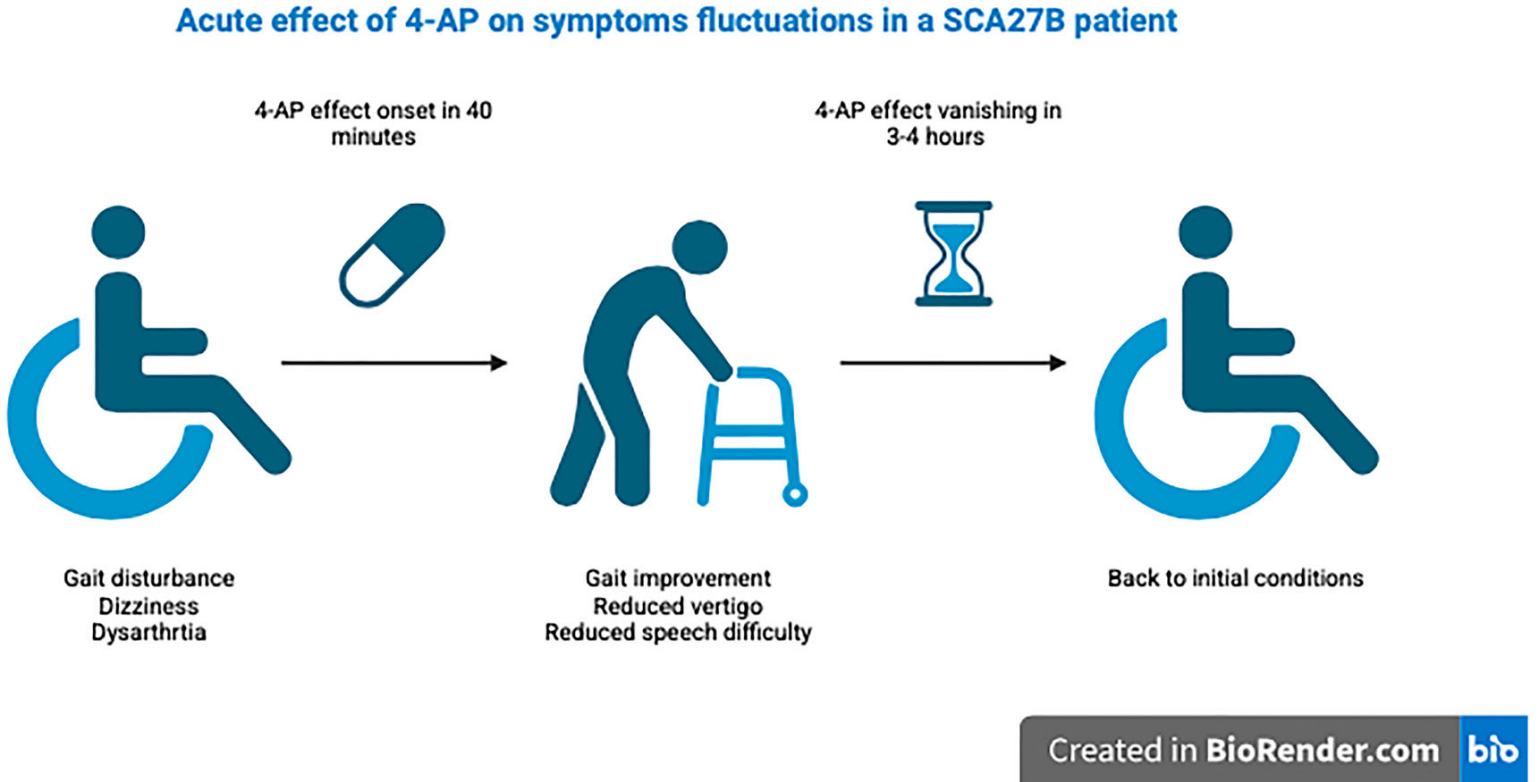
Graphical representation of treatment fluctuations with 4‐AP in our SCA27B patient.

**Video 1:** Downbeat nystagmus at baseline (morning, prior to 4‐AP intake; segment 1), and during the ON phase (40 minutes after 4‐AP administration; segment 2).

**Video 2:** Dysarthria at baseline (morning, prior to 4‐AP intake; segment 1), during the ON phase (40 minutes after 4‐AP administration; segment 2), and during the OFF phase (3.5 hours after 4‐AP administration; segment 3).

This observation provides two clinically relevant considerations. First, it supports the role of 4‐AP as symptomatic therapy in SCA27B, in line with its established use in other cerebellar disorders such as episodic ataxias (EA2)^5^ and downbeat nystagmus.^6^ Second, although response to 4‐AP is variable, with real‐world data indicating that approximately half of patients may not experience clinically meaningful benefit,^7^ a positive response may still represent a supportive clinical clue in patients with a phenotype suggestive of FGF14‐related ataxia. However, as responsiveness is neither universal nor disease‐specific, it should be interpreted cautiously and within the broader clinical context. Importantly, lack of response should not be interpreted as evidence against the diagnosis.

**Video 3:** Timed Up and Go (TUG) test at baseline (morning, prior to 4‐AP intake; segment 1), during the ON phase (40 minutes after 4‐AP administration; segment 2), and during the OFF phase (3.5 hours after 4‐AP administration; segment 3).

**Video 4:** Gait at baseline (morning, prior to 4‐AP intake; segment 1), during the ON phase (40 minutes after 4‐AP administration; segment 2), and during the OFF phase (3.5 hours after 4‐AP administration; segment 3).

**Video 5:** Finger‐to‐nose test at baseline (morning, prior to 4‐AP intake; segment 1), during the ON phase (40 minutes after 4‐AP administration; segment 2), and during the OFF phase (3.5 hours after 4‐AP administration; segment 3)

Consistent with previous reports demonstrating symptomatic benefit of 4‐AP in SCA27B,^2^, ^3^ our case further refines this evidence by documenting a clear, acute, and reproducible ON/OFF effect, highlighting the temporal dynamics of treatment response at the individual level.

The molecular mechanism is plausible, as *FGF14* encodes fibroblast growth factor 14, an intracellular protein modulating voltage‐gated sodium channels expressed in Purkinje neurons and thus regulating their rhythmic firing activity.^2^ 4‐AP, by blocking mainly Kv1 (A‐type) potassium channels, compensates the firing deficit of Purkinje cells and restores their function. The acute, reversible, and reproducible nature of the effect observed in this case highlights the clinical relevance of 4‐AP responsiveness in selected patients, supporting its role as symptomatic therapy and as a clinically informative feature within the broader diagnostic context.

To our knowledge, this is among the first reports to document a within‐subject, dose‐dependent and temporally reproducible improvement in a patient with genetically confirmed SCA27B treated with 4‐AP. Controlled studies are needed to clarify efficacy, ideal dosing, long‐term safety, and whether pharmacological challenge paradigms may have a supportive role within the diagnostic work‐up for cerebellar ataxias with episodic or fluctuating phenotypes.

## Authors Roles

(1) Research Project: A. Conception, B. Organization, C. Execution; (2) Manuscript Preparation: A. Writing of the first draft, B. Review and Critique.

C.C.: 1A, 1B, 1C, 2A, 2B.

E.B.: 1A, 1B, 1C, 2A, 2B.

A.P.: 1A, 1B, 1C, 2A, 2B.

S.S.: 1A, 1B, 1C, 2B.

F.M.S.: 1A, 1B, 1C, 2B.

M.T.: 1A, 1B, 1C, 2B.

I.A.D.V.: 1A, 1B, 1C, 2A, 2B.

## Disclosures

**Ethical Compliance Statement:** Ethical guidelines were followed in the absence of an institutional review board or ethics committee approval. The patient provided written informed consent for publication, including the use of video material. We confirm that we have read the Journal's position on issues involved in ethical publication and affirm that this work is consistent with those guidelines.

**Funding Sources and Conflicts of Interest:** None.

**Financial Disclosures for the Previous 12 Months:** None.

## Financial Disclosures and Conflicts of Interest

Author disclosures are available in the Supporting Information.

## Supporting information


**Data S1.** Supporting Information

## Data Availability

The data that support the findings of this study are available on request from the corresponding author. The data are not publicly available due to privacy or ethical restrictions.
